# Edible Films and Coatings Functionalization by Probiotic Incorporation: A Review

**DOI:** 10.3390/polym12010012

**Published:** 2019-12-19

**Authors:** Oana L. Pop, Carmen R. Pop, Marie Dufrechou, Dan C. Vodnar, Sonia A. Socaci, Francisc V. Dulf, Fabio Minervini, Ramona Suharoschi

**Affiliations:** 1 Department of Food Science, University of Agricultural Sciences and Veterinary Medicine, Calea Mănăştur 3-5, 400372 Cluj-Napoca, Romaniacarmen-rodica.pop@usamvcluj.ro (C.R.P.); dan.vodnar@usamvcluj.ro (D.C.V.); sonia.socaci@usamvcluj.ro (S.A.S.); 2 USC 1422 GRAPPE, INRA, Ecole Supérieur d’Agriculture, SFR 4207 QUASAV, 55 rue Rabelais, BP 30748, 4900 Agnes Cedex 01, France; m.dufrechou@groupe-esa.com; 3 Department of Biochemistry, University of Agricultural Sciences and Veterinary Medicine, Calea Mănăştur 3-5, 400372 Cluj-Napoca, Romania; francisc_dulf@yahoo.com; 4 Department of Soil, Plant and Food Science, University of Bari Aldo Moro, 70121 Bari, Italy

**Keywords:** edible films, edible coatings, probiotics, functional food, antibacterial activity

## Abstract

Edible coatings and films represent an alternative packaging system characterized by being more environment- and customer-friendly than conventional systems of food protection. Research on edible coatings requires multidisciplinary efforts by food engineers, biopolymer specialists and biotechnologists. Entrapment of probiotic cells in edible films or coatings is a favorable approach that may overcome the limitations linked with the use of bioactive compounds in or on food products. The recognition of several health advantages associated with probiotics ingestion is worldwide accepted and well documented. Nevertheless, due to the low stability of probiotics in the food processing steps, in the food matrices and in the gastrointestinal tract, this kind of encapsulation is of high relevance. The development of new and functional edible packaging may lead to new functional foods. This review will focus on edible coatings and films containing probiotic cells (obtaining techniques, materials, characteristics, and applications) and the innovative entrapment techniques use to obtained such packaging.

## 1. Introduction

Edible films or coatings (edible packaging or EP) are defined by any material meant to be applied (wrapping or coating) to food in order to extend the shelf life and may be consumed together with the food. Due to the many disadvantages related to plastic films and packaging, edible films have gained popularity in the scientific world, and drawn the attention of authorities and consumers [1] concerned about environmental protection. Indeed, conventional synthetic packages have a very damaging effect on the environment [2].

EP, especially those containing microorganisms, can be considered as a living ecosystem that selectively allows for the exchanges of respiration gases (e.g., oxygen, carbon dioxide and ethylene) between food and the atmosphere, diminishes or prevents loss of moisture and aromas and/or protects against undesired microorganisms [3].

Depending on the exact purpose, the film/coating can totally coat the food or can be applied between food constituents [4]. The materials that are utilized for the edible films/coatings production are biopolymers, proteins, lipids or composites. Thus, even if they are not consumed with food, they can be more rapidly and easily degraded with respect to plastic materials [5].

The main difference between coating and film is in their preparation and application process. Indeed, edible films are usually obtained in parallel to food and then applied to the surface, whereas coatings are directly prepared on food surface [6]. Both coatings and films can entrap live probiotic microorganisms.

Due to handling and hygienic limitations, EP can be combined with ecofriendly non-EP [6,7,8].

The utilization of films for food preservation dates back to the 12th century in China, where wax was utilized to delay moisture loss from fruits. Sixteen centuries ago, the first edible films made from soymilk were used in Japan for fruits preservation and in order to obtain a shiny surface [9,10]. Due to the narrow variety of materials used to protect fruits and vegetables at that time, no big interest was shown to this type of package. Refrigeration, controlled/modified atmosphere, heat or radiation sterilization, smoking have ever received stronger attention than edible packaging. Of course, food conservation methods have considerably increased and have offered unlimited opportunities to prepare, store and consume any type of food in any season. However, EP can currently be applied to a large variety of food products, with unique, tailored and innovative ways of action than conventional food preservation techniques [1].

Among various roles played by EP, physical protection [11] amplification and protection of food properties, carriers of food additives and prolongation of shelf life are the most important ones.

EP may be categorized according to the class of their constituent material. Hydrocolloids (polysaccharides and proteins) and lipids are the most used materials. Among these, polysaccharides are the easiest to purchase and more suitable to form films or coatings. The presence of a large number of hydroxyl groups and hydrogen bonds favor the formation of film. Different properties can be observed between films and coatings made of negatively charged gums (i.e., alginate, pectin, or carboxymethyl cellulose) [7].

Proteins used for EP have mostly animal origin (gelatin, casein, whey proteins, collagen or egg albumin). However, plant-derived proteins (e.g., corn, soybean, wheat, cottonseed, peanut, and rice) are also appreciated and compatible with the vegetarian diet. The film/coating forming process is started, in most of the cases, with protein denaturation using heat or pH adjustment, followed by a conglomeration of peptide chains through new intermolecular interactions [12]. These type of films are suitable mainly for meat products, due to their affinity to hydrophilic surfaces [13,14,15].

Lipids do not form cohesive films, unlike hydrocolloids. For this reason, they are used especially for coatings or in mixture with polysaccharides in order to obtain an optimized water vapor barrier [16].

The integration of different additives (i.e., probiotic microorganisms but also organic acids [17], essential oils [18], plant extracts [19], and antibacterial compounds [20]) into the EP has the benefit of ensuring slower release of these compounds to food [21]. The aim of this paper is to review the application of various types of natural EP that incorporates live probiotic microorganisms.

## 2. Bioactive Molecules in EP and Perspectives in Food Industry

EP may be used not only for their protective effect but as carriers of bioactive substances too. Some examples of bioactive molecules are: antimicrobial compounds, probiotics, anti-browning compounds, omega-3 fatty acids, and other nutraceuticals [22]. Active food packaging that incorporates bioactive molecules not only acts as a traditional protective system for the food product, but also promotes the health of consumers.

Even more, the utilization of byproducts in order to obtain the edible package or to extract a bioactive molecule that will be further incorporated into the package will sustain the economical approach [23]. Obviously, when compared with fresh fruits and vegetables, the utilization of agricultural byproducts, like fruit peels to prepare edible films, seems much more profitable from the perspective of resource recycling and environmental protection, and needs further study [24].

The utilization of EP that contain bioactive molecules have multiple advantages [25]. One example of EP that can sustain human health and influence the final product are fried food products. A decrease of oil intake in deep-fried food products represents a significant application of EP, namely coatings. Obesity and heart diseases have been linked to an excess of fat in food. The use of film made of methylcellulose and hydroxypropyl/methylcellulose allows for a decrease in oil absorption by food, thus helping to reduce fat intake [26].

The processing techniques used to obtain EP vary depending on the material and bioactive compounds added in the EP [27]. Legislation, polymer types, active molecules, destination and are all factors that influence the matrix choice.

## 3. Probiotics in Food and Human Health

Nowadays, probiotics are associated with a world that lives on and inside humans and animals, modulating the host’s health [28,29]. The amount of bacteria that can be found in the human body exceeds the number of human cells by more than ten times. Due to the great impact of the gut microbiota on the human body and its health modulation, the human gastrointestinal network is also called “the other brain” [30]. The human gut hosts about 1500 bacterial species, of which about 500 species have pathogenic or probiotic traits.

The gut’s influence on health, exerted by microorganisms inhabiting human body (especially some sections of the gut), starts in the womb of mother, depends on the child’s delivery (C-section or vaginal) modality, milk (breast-fed or artificial milk) ingested by newborn, and afterwards, is mostly modulated by diet. Other genetic and epigenetic factors, as well as environmental drivers (geographic location, stress, physical activity, and drug intake), further modulate the balance in the gut microbiota. While being relatively stable in adulthood, during aging, the gut microbiota composition continuously changes [31]. In elderly individuals, the frequently observed decrease in the *Bacteroidetes*/*Firmicutes* ratio is correlated with functionality decline of the immune system.

Any modification in the diversity of the gut microbiota (dysbiosis) may result in the onset of certain illnesses and dysfunctions. The use of probiotic supplements is a possible, cost-effective and easy-to-use solution to counteract dysbiosis and face the pressing issue of microorganisms capable of resisting multiple antibiotics [32].

The current definition of probiotic microorganism underlines it as a viable, single or mixed, culture of bacteria or yeast which beneficially impact animal or human health when ingested in the adequate amount [33]. Members of the genera *Bifidobacterium* and *Lactobacillus* are consistently used for their probiotic effect, whereas members of the genera Streptococcus and Enterococcus contain several opportunistic pathogens [34,35]. Some yeasts, mostly *Saccharomyces boulardii*, are accepted for use as probiotics.

Probiotics help to prevent or, in some cases, treat diarrhea, ulcerative colitis, irritable bowel syndrome, allergies, obesity, and diabetes [36,37]. Several modes of action are well-known for probiotics; for instance, they are able to modulate nutrients absorption [38], act as a barrier against pathogenic bacteria at the level of intestinal mucosa [39], have an impact on the immune system [40], and influence the gut–brain axis [41]. Some mechanisms of action exerted by probiotic microorganisms are mediated by their metabolites, such as molecules with antimicrobial activity (e.g., organic acids, ethanol, hydrogen peroxide, and bacteriocins) and short chain fatty acids that are used by enterocytes as nutrients [42].

## 4. Entrapped Probiotics in EP

**Figure 1:**
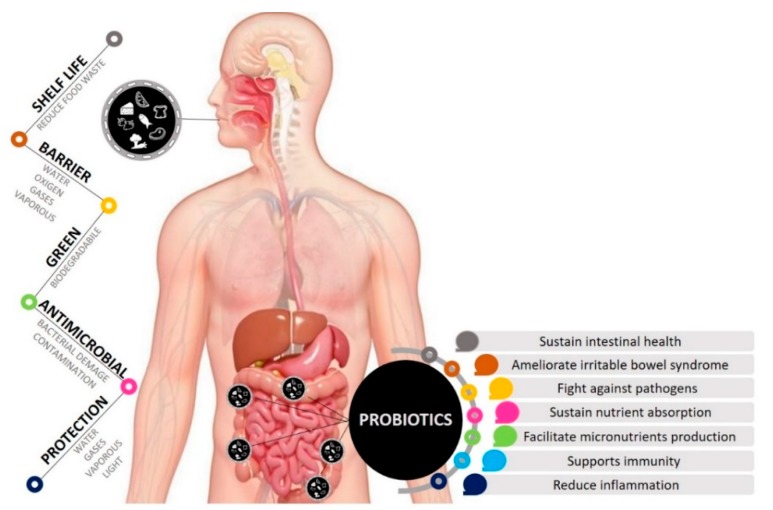
Characteristics of edible packaging (EP) containing probiotics and some of the most studied healthy effects exerted by probiotics.

In order to benefit from the consumption of probiotics, a dose of 10^8–9^ viable cells per day is recommended. In many products, to reach this dose is challenging due to high sensibility of probiotics to environmental conditions. Survival of probiotics depends on strain, food characteristics (e.g., pH), processing technologies, storage conditions and time [43]. Biological activities of probiotic bacteria and yeasts can be negatively affected by their loss of viability during food processing and storage. The use of encapsulated probiotics in edible films or coatings could favor the optimal survival of beneficial microorganisms in food. EP that incorporate probiotics display, besides those characteristics that are peculiar to all the EP, features specifically addressed to maintain the host in good health (Figure 1). In addition, since probiotic microorganisms often showed inhibitory activity towards spoilage or pathogenic bacteria, their incorporation in EP can increase food stability and safety. Food packaged in coatings or films containing probiotics may be regarded as functional food, a special group of food items that, if regularly introduced in diet, benefit health, beyond their nutritional value [44].

Encapsulation of probiotics in EP may be obtained using spray drying [45] with or without protectants, spray freeze-drying or electrospray, and cross-linking gelation.

The addition of probiotics to EP is much less frequent than the addition of plant extracts. Nowadays, this technology allows widening the range of probiotic-carrier food products that vehicle probiotics, satisfying the demand for nondairy foods, fostered by vegan consumers and lactose-intolerant people [40]. In one of the first research studies about the encapsulation of probiotics in EP, *Bifidobacterium animalis* subsp. *lactis* BB-12 was entrapped in alginate and gellan-based edible coatings of apple and papaya slices. The addition of BB-12 seemed to cause an increase in the space between the polymer chains. During 10 days of storage at 2 °C, the cell density of the strain was above minimum recommended (10^6^ Colony-Forming Units/g or CFU/g) [46,47]. However, the coating containing the probiotic strain showed higher (50%) water vapor permeability than the control coating [46].

EP containing probiotics could be exploited to overcome the otherwise unavoidable loss of viability of beneficial microorganisms during food processes carried out at high temperatures. Microcapsules containing a probiotic strain of *L. acidophilus* were dispersed (1% or 2%) in a starch (5%) solution, which covered the surface of bread dough [48]. This technology allowed *L. acidophilus* to keep its viability after baking, without any negative impact on the taste of bread and texture properties of the crumb. In addition, the edible coating reduced bread crust crispness [48].

## 5. Materials and Techniques Used for Probiotic EP

**Figure 2:**
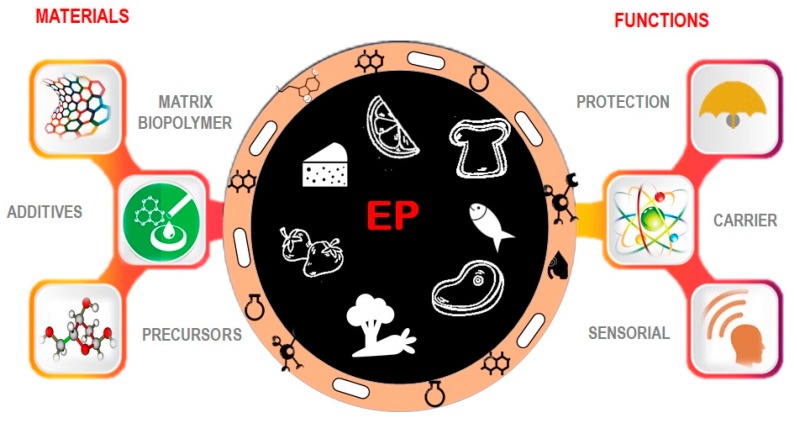
Edible films and coatings components and roles.

Commonly, EP are expected to be transparent, flavorless and unable of modifying the sensory properties of food products. However, some applications (e.g., sushi wraps) may require specific sensory properties as lack of evolution of negative organoleptic characteristics. EP are usually composed of two major components: (i) a macromolecule-based substance, biopolymers, (ii) additives as plasticizers, cross-linkers, nanoreinforcements and (iii) precursors as proteins, polysaccharides, lipids or resins (Figure 2). The macromolecule-based substance represents the base that, dissolved in a solvent (usually water), forms a cohesive assembly. The plasticizer is added in order to improve mechanical properties of package (e.g., elasticity, toughness, resistance to tearing), so that the package gains flexibility and higher stability [14]. Plasticizers, such as sorbitol, polyethylene glycol, glycerol and sucrose, are commonly needed when the package is composed of proteins and polysaccharides. In some cases, emulsifiers are used, instead of plasticizers, in order to increase the stability of film/coatings, made of lipids and polysaccharides [49]. Due to the materials and/or due to the incorporated active molecules, the EP are meant to protect the food or just to act as a carrier for the active compounds, to reduce contamination, to improve/maintain the food product natural appearance, to enhance the mechanical properties of fragile food products or to boost the appearance and flavor (Figure 2).

Diverse biocompatible components, such as hydrocolloids, lipids and composites, are used in EP preparation [50]. According to their specific purpose, miscellaneous compounds may be exploited for entrapment of probiotics in EP are miscellaneous. In these cases, the package is defined as composite [5,16].

Hydrocolloids include polysaccharides and proteins. Among polysaccharides, cellulose and its derivatives, dextran, inulin, alginate, carrageenan, starch derivatives, pectin derivatives, chitosan, seaweed extracts, and galactomannan are the most utilized for edible films and packages [14,24,51]. All polysaccharides successfully protect food from oxygen, odor, and oil absorption; on the other hand, they show high water permeability [49]. In subsequent paragraphs, a concise presentation of the most utilized materials is made:

Cellulose and cellulose derivate (e.g., methylcellulose and hydroxypropyl methylcellulose) prevent oil absorption from fried food items [52] and have been successfully used for EP-containing probiotics [53,54,55]. Alginic acid, also known as alginate, may be conveniently applied to meat products, where it considerably delays lipid oxidation [56,57,58]. Chitosan is obtained from chitin deacetylation and is usually obtained from the exoskeleton of crustaceans and fungal cell walls [59]. The deacetylation process influences the chitosan molecular weight and, in turns its properties (i.e., crystallinity, hydrophobicity, degradation, tensile strength and moisture content) [60,61]. Chitosan shows antimicrobial properties [62,63]. Starch and its derivatives are cost-effective and easy to handle. In addition, they are typically clear, inodorous and insipid [64,65]. The starch films and coating characteristics are strongly influenced by the amylose/amilopectin ratio. A strong and flexible film is obtained from a starch rich in amylose content [66]. Pectin, frequently utilized in jams and jellies, was used to produce films and coatings containing probiotics [67,68].

Proteins are dissolved or dispersed in solvents (i.e., water or ethanol) that are further evaporated in order to obtain the package. The protein-based structure forming process is favored by heat or acid conditions [69,70]. Compared to polysaccharides, proteins have lower vapor permeability.

Lipids may be commonly utilized in the form of waxes, oils, fats, and resins for building EP-entrapping probiotics [71]. Since they are characterized by a high resistance to water penetration, in most cases, the lipids are combined with polysaccharides or proteins [72].

Table 1 summarizes some of the types of composite used in order to obtain EP, the materials and the designated food products with some generic and specific effects.

The materials used for EP may be derived from food industry byproducts, such as whey, corn zein (source of proteins), mung bean or fruit pomace (source of pectin). This represents an environmentally friendly solution and assists in cost reduction. Nevertheless, the utilization of food byproducts in EP could signal consumer mistrust due to confusion between byproducts and wastes.

Extensive applications of mentioned materials has been literally obstructed by some difficulties in the material preparation process [73,74]. Most of these difficulties are related to the solubility of the materials in solvents that are accepted in food industry. However, scientists innovate in order to obtain best properties of the EP. An edible biocomposite film was proposed to be obtained directly from psyllium seed, but it was proven that the utilization of seeds husk and husk flour was more suitable [75]. In general, lipids are difficult to apply on the surface of some foods due to their poor adhesion to food products with hydrophilic surfaces [76]. Chitosan can ensure many benefits, such as excellent hydrophilicity, high porosity, big adhesion area, and can be cross-linked to avoid dissolution in acidic solutions (pH < 2). The use of chitosan as material for the entrapment of probiotics has been widely studied, but the too-soft texture and similarities between the density of the EP and that of water (leading to easy float) limits its industrial function. Therefore, efforts have been made to support the structure through the addition of activated clay and crosslinking with glutaraldehyde, which has been demonstrated to permit superior operational stability. However, these alternatives are not suitable for the food industry. Nevertheless, more studies regarding the challenges in the materials preparation process need to be conducted in order to smooth the processes and sustain this environmentally friendly method [77].

In order to sustain the applicability of probiotic EP in the food industry at an industrial scale, new and innovative techniques need to be developed. Nanotechnology and the utilization of nanomaterials is a promising area that can broaden the use of probiotic EP. Formulation of non-nanomaterials in nanosized structures can bring enormous benefits due to the new and unique obtained bioactive properties [78]. The utilization of electrospinning in the preparation of EP materials can be a suitable technique for the restructuration of biopolymers in nanoscale.

## 6. Probiotics Viability in EP

Probiotics may be used in pharmaceutical or food-based products [99,100]. The edible coating or films may be regarded as a carrier of probiotics. The major challenge to be faced by probiotic microorganisms is their resistance to entrapment, an essential prerequisite for their viability in the final product. Only viable probiotics at adequate cell numbers can successfully colonize the colon. Some studies were specifically devoted to investigating the viability of probiotics entrapped in edible coatings/films. Composition and storage temperature affect viability of probiotics in edible coatings/films. Pullulan is a polysaccharide that can be used as a base for EP. A pullulan-based film embedding probiotic lactobacilli (*L. reuteri* ATCC (American Type Culture Collection) 55730, *L. rhamnosus* GG ATCC 53103, and *L. acidophilus* DSM 20079) proved to sustain the viability of probiotics, during 10 and 20 days’ storage at room temperature at levels of 10.3 and 4.5 log CFU/mL, respectively. A similar film, but containing a mixture of pullulan and potato starch, was less effective in maintaining the viability of probiotic lactobacilli. In detail, the higher the starch content, the lower the probiotic viability. However, when lower storage temperature (4 °C) was applied, no differences were found in terms of viability loss between the pullulan- and pullulan/starch-based film. The viability loss did not exceed 10% even after 30 days of storage [101]. Entrapment of *L. rhamnosus* GG in a film based on a mixture of starch (from rice and corn) and proteins (bovine gelatin, sodium caseinate, and soy protein) resulted in higher viability of the probiotic strains at refrigeration than at room temperature. The presence of proteins increased viability of *L. rhamnosus* GG during the film formation process [102]. *L. plantarum* and *Kluyveromyces marxianus* incorporated in a film composed of kefiran (a polysaccharide secreted by lactic acid bacteria) and glycerol did not negatively affect the film optical and physical properties. Compared to a suspension, both microorganisms showed better tolerance to acid conditions in the film and maintained their viability through storage at room temperature. In addition, the yeast showed higher resistance to the film-forming procedure than the lactic acid bacterium [103]. *B. animalis* subsp. *lactis* BB-12 was incorporated in alginate and gellan (2% solutions) edible coatings and applied on fresh-cut apple and papaya. Although a viability decrease of the probiotic higher than 85% was observed, BB-12 was maintained above the minimum recommended (10^6^ CFU/g) [46].

## 7. Synbiotics in EP

Probiotics may be combined with prebiotic compounds, i.e., substances capable of favoring beneficial microbes in the human gut. The term “synbiotics” is used for indicating products containing at least one probiotic microorganism and one prebiotic substance. Such products may help to maintain the cell viability of probiotics and have been experimented inside edible films. The presence of fructooligosaccharides (FOS) as prebiotic compounds in a methylcellulose-based film containing two probiotic strains (*L. delbrueckii* subsp. *bulgaricus* CIDCA 333 and *L. plantarum* CIDCA 83114) was effective in the protection of both probiotics. However, it had a negative effect in the film forming-process, by reducing the glass transition temperature of the film [66]. Inulin, galacto-oligosaccharide and FOS in chitosan-based film favored viability of probiotic *Bifidobacterium infantis* ATCC 15697 and *Lactobacillus fermentum* ATCC 9398. Besides the prebiotic effect, the oligosaccharides increased the extensibility of the film, compared to a prebiotic-free film [104]. Viability of *L. rhamnosus* GG was monitored during time in a gelatin-based film added with inulin, polydextrose, gluco-oligosaccharides and wheat dextrin. The presence of prebiotic compounds did not impair the film structure. Viability loss was found regardless of the type of prebiotic compound, but especially with film containing gluco-oligosaccharides (about 40%) or polydextrose (almost 85%). Among the tested prebiotics, inulin allowed to maintain viability of the probiotic strain at acceptable level over 100 days of storage, whereas in the film containing the other compounds an acceptable viability was maintained for a shorter time (63–83 days) [102].

Thus, the limitations and difficulties in the utilization of pro- and prebiotics in EP formulations need to be addresses, despite the fact that very few scientific papers discuss this aspect. The utilization of prebiotics, together with the probiotics may lead to serious changes in the final properties of EP. Ensuring low production costs is the main challenge for future EP process and formulation technologies. The exploitation of food-grade raw materials such as native, and physically or enzymatically processed biopolymers, is one example of a method that has the potential to meet the challenge of widening the range of EP types into which probiotic and prebiotic can be favorably incorporated [105]. Novel developments for control release systems from the EP will also provide new possibilities. Negative changes in the EP formulation, that are not affecting the characteristics of food products and ensure the extension of shelf life (i.e., transparency, brightness, etc.) will be accepted by the consumers only if they realize that the benefits related to the presence of prebiotics in the probiotic EP are greater than the characteristic related to the food appearance.

## 8. Antimicrobial Effects of Probiotics Incorporated in EP

Besides their positive effects on human health, probiotic microorganisms incorporated in EP could protect food from pathogenic bacteria, leading to increased food safety. They could also inhibit spoilage microorganisms, thus extend the shelf-life of food. A probiotic strain belonging to *Lactobacillus sakei* was embedded in a sodium caseinate-based film through either direct incorporation in the film-forming suspension or by spraying on an already-formed film. The film, and its counterpart not containing probiotic lactobacilli, were applied on plates of tryptic soy agar on fresh beef slices, which were inoculated with *Listeria monocytogenes*. During four days of incubation on plates, the probiotic strain increased of one log cycle its cell density. *L. monocytogenes* decreased (3.0–3.6 log cycles) during the 12 days of storage. In the beef slices stored at 4 °C for 21 days, *L. sakei* cell density was higher than 6 log CFU/cm^2^. In addition, the cell density of the pathogenic bacterium was two log cycles lower than in the probiotic-free film [106]. In the presented study, it can be observed that the presence pf probiotics from lactobacillus species negatively influenced the multiplication of *L. monocytogenes* on the beef slices by producing bacteriocin-like substance. Thus, the production of this substance was nonexistent after a long period of time. This fact can be explained by the death of *lactobacillus* as an effect of the environmental conditions and lack of nutrients.

A similar study that echoes the above-presented results is an alginate-based film containing *Carnobacterium maltaromaticum*, a potential probiotic bacterium normally found as commensal of various fish species [107,108], was applied on smoked salmon, inoculated with *L. monocytogenes* at 4 log CFU/cm^2^. This film had a bacteriostatic effect towards *L. monocytogenes* during 28 days of storage at 4 °C [109]. The authors of the study declare that the antibacterial effect can be explained due to the neutralized supernatant and therefore was not due to acidity or pH.

A gelatin-based coating containing probiotic strains of *L. acidophilus* and *Bifidobacterium bifidum* was applied to hake (*Merluccius merluccius*). Both probiotic strains maintained their initial level (10^9^ CFU/mL) of viability for 6 days of storage at 2 °C. The spoilage agent *Shewanella putrefaciens*, typically producer of H_2_S, was found in coated hake at significantly lower counts than the uncoated hake. However, the antibacterial effect had no relevant link to the presence of probiotics in the edible package. Treatment of coated hake through high hydrostatic pressure (200 MPa for 10 min at 20 °C) proved to be effective in decreasing the spoiling agent, but had no effect on the viability of probiotics [98].

The ability of an agar-based film, incorporating green tea extract and two probiotic strains (*Lactobacillus paracasei* L26 and *B. animalis* subsp. *lactis* B94), to inhibit two spoiling bacteria was investigated in hake fillets. The spoiling agents, *S. putrefaciens* and *Photobacterium phosphoreum* were deliberately added (10^3^–10^4^ CFU/g) to hake fillets, prior to film application. The results showed that probiotic bacteria migrated from the film to fish and that fish wrapped in the film displayed lower values of spoilage indicators compared to untreated fish (e.g., pH, count of H_2_S-producing bacteria, concentration of trimethylamine). Overall, the use of this probiotic film extended the shelf life of hake fillets for at least one week [110].

The type of material constituting the edible package affects probiotics viability and their antimicrobial activity. A probiotic strain of *L. plantarum* was embedded in an edible film based on sodium caseinate, pea protein, methylcellulose or hydroxymethylcellulose [54]. The probiotic strain showed higher viability in protein than in cellulose-based film. Interestingly, when applied in the cellulose-based film, *L. plantarum* produced higher levels of bacteriocin, resulting in the total inactivation of *Listeria innocua* during 8 days of storage at refrigerated temperature [54].

When incorporated in sodium caseinate- or methylcellulose-based film, *L. acidophilus* displayed higher viability than *L. reuteri*. After three days of storage, higher antilisterial activity was found for the methylcellulose-based film than for the one made of sodium caseinate. Compared to similar films without probiotic lactobacilli, *Listeria* sp. decreased by about 1.5 log cycle after 12 days of storage [111].

Alginate, whey proteins, or a mixture thereof were used for forming an edible coating containing *L. rhamnosus* GG and was applied to bread [43]. During the two drying processes considered (60 °C for 10 min, 180 °C for 2 min), the composite-based coating provided *L. rhamnosus* GG with higher protection, with respect to alginate- or whey proteins-based coating. However, following simulated gastrointestinal digestion, the highest cell density of *L. rhamnosus* GG (10^6^ CFU/g) was found in the bread coated with alginate [43].

The antibacterial activity of probiotics embedded in EP is limited due to the specific activity of the probiotic metabolites. This fact can explain why same probiotic strain act as antimicrobials against certain pathogens and some have no influence. Nevertheless, as seen [54], the material used for the incorporation of probiotics has a great impact regarding the antimicrobial activity of the probiotic strain. This activity modulation can be correlated to the permeability of the EP for the antimicrobials metabolites produced by the probiotic cells and by the material capacity to protect the active cells.

## 9. Concluding Remarks

Nowadays, the increasing consciousness of consumers about the link between dietary habits and health fosters the market of food containing probiotic microorganisms. EP technologies allow us to broaden the fields of application of probiotics to unexplored food items (e.g., baked goods). Overall, at an industrial scale, the number of applications of edible coatings/films containing probiotics is much lower than that of research studies carried out in the laboratory. One of the major challenges to be faced in order to achieve a wider industrial application is to obtain the perfect combination of materials, technologies and probiotic strains, tailored to specific foods and consumers’ needs, and at an acceptable cost. Another challenge is in the need to maintain a high cell density of probiotics during the formation process of EP and, especially, after ingestion. This is a prerequisite to impact human health positively. Future research efforts should be dedicated to these two challenges. In addition, a higher number of studies about the health benefits of EP are essential.

## Figures and Tables

**Table 1:** Some polysaccharides, proteins, lipids, and composites-based EP for different food products with their generic and specific functions.

Materials/Methods		Generic Effects	Specific Composition	Type of Food	Specific Effects	Reference
Polysaccharides	Starch	+ colorless + oil-free appearance + reduced caloric content + prolong shelf life + suitable for fruits, vegetables, meat + control oxygen transmission + reduce darkening of the surface - no moisture barrier - hydrophilic nature	Starch-based coatings with D-glucose, silver nitrate.	Chicken Sausages	Antimicrobial activity.	[79]
	Cellulose and derivatives		Hydroxypropyl methylcellulose (HPMC) and beeswax coatings.	Cherry tomatoes	Prevent weight loss, sustain fruit firmness, improved sensory attributes.	[80]
	Pectin		Pectin and sodium alginate coatings with citral and eugenol essential oils.	Raspberries	Maintain the color, prevent weight loss, trolox equivalent antioxidant capacity, prevent microbial growth.	[81]
	Pullulan		Pullulan-based coatings with sweet basil extract.	Apples	Sustain color, appearance and sensory attributes during hypothermia storage.	[82]
	Alginates		Alginate - chitosan and ZnO nanoparticle	Guavas	Increase the shelf-life of the fruit.	[83]
	Chitosan		Chitosan-based coatings with vacuum packaging.	Beef	Effects on color preservation and lipid oxidation during retail presentation.	[84]
Proteins	Vegetable-based proteins	+ provide mechanical stability + good transparency - not suitable for some diets (vegan)	Whey proteins coatings with lysozyme.	Salmon	Overall quality of salmon.	[85]
			Gluten and zein coatings with potassium caseinate, rennet casein, xanthan gum, locust bean additives.	Trout Fillets	Sensorial attributes and the physical biochemical qualities.	[86]
	Animal-based proteins		Caseinate-based coatings with ascorbic acid additives.	Beef	Effect of gamma irradiation on microbiological characteristics of ground beef.	[87]
			Furcellaran-gelatin-based edible coating.	Salmon sushi	Exhibit good transparency, mechanical and barrier properties and can be manufactured by extrusion or casting processes.	[88]
Fats	Oils	+ reduce water transmission	Lipid-based (sunflower oil and chocolate) coating with stearic acid, polyglycerol.	Apple slices	Moisture barrier.	[89]
	Waxes		Candelilla wax coating with ellagic acid.	Avocado	Antifungal characteristics to enhance shelf life.	[90]
			Carnauba wax coating.	Eggplant	Increase in the water vapor resistance and reduction in weight loss.	[91]
			Candelilla wax coatings with mineral oil.	Guava fruit	Weight loss ethylene emission, gloss, retention of the color, firmness.	[92]
			Chitosan-Beeswax coating.	Strawberries	Reduction in weight loss.	[93]
Multicomponents/Composites		+ special tailored for specific characteristics + enhance the permeability or mechanical properties - may get expensive	Composites of carrageenan and whey protein coatings with CMC sodium salt, polyethylene glycol, calcium chloride, glycerol and oxalic acid additives.	Apples	Reduce brownness.	[94]
			Composite of chitosan and gelatin coatings.	Red bell peppers	Improve firmness, diminish weight loss, and ethanol concentration.	[95]
			Composite of hydroxypropyl methyl cellulose (HPMC) and lipid coating with potassium sorbate, sodium benzoate, sodium propionate, stearic acid, glycerol additives.	Oranges	Antifungal properties improved during long-term cold storage.	[96]
			Shellac, gelatin and Persian gum.	Orange	Improve permeability characteristics.	[97]
			Hydroxypropyl methylcellulose-lipid composite edible coatings.	Citrus fruits	Maintain postharvest quality.	[98]
